# Perceptions of Close Relationship Through the Machiavellians´ Dark Glasses: Negativity, Distrust, Self-Protection Against Risk and Dissatisfaction

**DOI:** 10.5964/ejop.v14i4.1550

**Published:** 2018-11-30

**Authors:** Tamás Ináncsi, Attila Pilinszki, Tünde Paál, András Láng

**Affiliations:** a Department of General and Evolutionary Psychology, University of Pécs, Pécs, Hungary; b Faculty of Health and Public Services, University of Semmelweis, Budapest, Hungary; c Independent researcher; d Department of Personality, Development and Clinical Psychology, University of Pécs, Pécs, Hungary; Department of Psychology, Webster University Geneva, Geneva, Switzerland; Institute of Psychology, University of Wroclaw, Wroclaw, Poland

**Keywords:** Machiavellianism, negative attitudes, distrust, self-protection, relationship dissatisfaction, actor-partner effects

## Abstract

It is commonly known from the literature that Machiavellian individuals have negative attitudes towards people and in general towards the world´s affairs. They are distrustful of the intentions of others, and they get cautiously involved into interpersonal interactions and take risks only if that may not have any severe negative consequence. It is also a fact that there are few ventures in life that potentially involve as much insecurity and personal vulnerability as the establishment and maintenance of close relationships. In our study, we were seeking the answer to the question: do people with high levels of Machiavellianism show a generally negative, distrustful and cautious attitude in their intimate relationships, as well? What effect their pessimistic approaches have on the other consequences of the relationship (satisfaction, commitment, investment, quality of alternatives)? This question was investigated on a dyadic sample of heterosexual couples (N = 101 pairs) with Actor-Partner Interdependence Model (APIM). The results of the correlations and actor effects show that men with high levels of Machiavellianism perceive in a negative way not just people in general, but their romantic partners and relationships as well and they experience an increased level of distrust, risk, and dissatisfaction into their close relationships. Women with high levels of Machiavellianism are less negativistic and feel less discontent towards their intimate partner and relationship, but even they are unable to put their distrust and precaution aside. The results of partner effects have revealed that women's Machiavellianism undermines men's trust, while men's Machiavellianism has the effect of minimizing women's investment into their relationship.

## Machiavellianism and Negativity Originating From Early Life Experiences

According to Christie and Geis (1970) there are people among us - the Machiavellians - who are mostly predisposed to gaining unjust benefits by trying to mislead, manipulate and exploit others. The psychological organization that grounds this operation is also manifested in close relationships and has a serious influence on the development of mutually dependent relationships, therefore it is especially important to investigate the Machiavellian psychodynamics also within the context of the relationships. Since it is impossible to understand the deeper connection of how adult relationships operate without ignoring childhood antecedents (Hazan & Shaver, 1987), this article looks on the romantic relationship functions of Machiavellian in a broader framework, without making childhood experiences part of the research design.

Based on the studies made so far, it seems that shared environmental factors have a greater impact on the development of Machiavellian personality trait than genetic factors (Vernon, Villani, Vickers, & Harris, 2008). Parents who are neglecting, psychologically unavailable, who lack warmth and provision of emotional security, or who are severely punishing or rejecting (Jonason, Icho, & Ireland, 2016; Kraut & Lewis, 1975; Láng & Lénárd, 2015; Ojha, 2007; Touhey, 1973), often have children with distorted internal object relations (Janoff-Bulman, 2010; Kernberg, 1975; Kohut, 1971) and early maladaptive schemes (Láng, 2015). This lack of optimal parenting could lead to developing a negative, pessimistic, distrustful, hostile, and cautious attitude in Machiavellian individuals (Rauthmann, 2012).

Since childhood attachment relationships form the foundation upon which all adult relationships are built (Shaver, Hazan, & Bradshaw, 1988), therefore representations of insecure, traumatic object relations experienced in early years of life usually continue to live in adult relationships as well. The representations of experiences of childhood grievances and failures may function as latent vulnerabilities and the frustrating contexts and objects may transfer from their past relationships to the present ones. In order to get rid of their tormented feelings, Machiavellian persons transfer their frustrating, bad objects (which may be self or object representation) along with their negative emotional state into other people and identify others afterwards with these inner contents (Richardson & Boag, 2016). Looking through the distorting glasses, they perceive others as malicious, hostile and threatening, and they condemn people to gain control over the situation and their own internal insecurities. In this self-object relationship the dominant emotions are anger, hatred, contempt, and desire for revenge (Ináncsi, Láng, & Bereczkei, 2015). These are the processes of transference and projection (projective identification), immature defense mechanisms often used by Machiavellians to cope with their anxieties (Richardson & Boag, 2016).

Machiavellian people are prone to remember negative past experiences and paint a gloomy, uncertain future that is predestined by fate (Birkás & Csathó, 2015). These results also suggest that Machiavellian attitude is not so much a voluntary choice as it is a pathway primed to the negative direction by the toxic childhood experiences.

## Machiavellianism and Intra- and Interpersonal Negativity

Consistently with the afore-mentioned, most studies found that Machiavellianism is positively associated with neuroticism (Douglas, Bore, & Munro, 2012; Jakobwitz & Egan, 2006; Muris, Meesters, & Timmermans, 2013; Paulhus & Williams, 2002; Vernon et al., 2008; Veselka, Schermer, & Vernon, 2012). As known from the literature (e.g. Costa & McCrae, 1980; Eysenck, 1987), neuroticism is a personality trait that predisposes to have negative emotional approach towards others and to assign them negative attitudes and unfavourable attributions (Robinson, Ode, Moller, & Gotez (2007), since the more important an emotional dimension is for the individual, the more likely the individual would evaluate the world based on this dimension (Shah & Higgins, 1997). At the same time, negative expectations also lead to more negative perceptions. Indeed, Machiavellian individuals are commonly viewed that they focus on the negative side of others and of the world in general, and perceive others in a negatively biased way (Rauthmann, 2012). In their cynical and misanthropic worldview, people are not good, not kind and basically untrustworthy (Christie & Geis, 1970). In Rauthmann's (2012) study, Machiavellian people saw others as less caring, less intelligent, and less socially skilled. In the same study, it is also pointed out that Machiavellianism is the most negativistic personality type within the Dark Triad [Machiavellianism, narcissism, and psychopathy (Paulhus & Williams, 2002)]. This is probably because the representational world of the Machiavellian individuals is organized along the splitting (Jonason et al., 2018). Since in their representational world, "bad objects" dominate, they are incapable of evaluating others in a “good and bad matrix” (Siegel, 1992, p. 55). They do not recognize that everyone has a good and bad side, which disorients them to see others realistically. Indeed, Richardson and Boag (2016) have demonstrated that Machiavellianism positively correlates with the defensive mechanism of splitting.

The Machiavellian people do not want to show themselves or their intentions better than they actually are. For outside observers, it seems that they do not feel their motivations and actions ego-dystonic. Jonason and Jackson (2016), however, found that Machiavellianism was positively correlated with negative feelings about the self. Machiavellian individuals are aware of their anomalous nature of their character, so they are less believing that others can truly love them. Machiavellian individuals see themselves as less caring, less open and less sociable (Rauthmann, 2012), thus devaluing the importance of the intrinsic relationships goals (McHoskey, 1999). Since the models of the self and of others are similar, and the perception of others is in relation to the self-image of the perceiver (Hoorens, 1996; Lewicki, 1983), Machiavellian individuals do not realize the feelings of communality in others. This result is consistent with Machiavellian individuals' expectations of an ideal partner, as Machiavellian people feel less important the ideal partner to be warm-hearted and loyal, since they themselves have no such features (Ináncsi, Láng, & Bereczkei, 2016). There is also evidence that Machiavellian individuals project their own insecure personality to people, preventing them from seeing the best in others. They rather seek and find other people's faults and attackable weaknesses than idealizing them in the positive direction (Richardson & Boag, 2016). Although no interpersonal relationship exists without mutual idealization and positive illusions (Murray, Holmes, & Griffin, 1996a, 1996b), it seems from the previous results that Machiavellians are less likely to associate their fellows with positive qualities because they do not even believe in the positive values.

Studies have also shown that Machiavellians are not accurate in assessing the characteristics of other persons, they show superficial, stereotypical, less differentiated judgments of others (Rauthmann, 2013), a "negative heuristics of others" (Black, Woodworth, & Porter, 2014, p. 52).

## Machiavellianism and Attitudes of Distrust

Most results indicate that Machiavellian individuals’ personality has been irreversibly injured due to the trauma they lived through in the early stages of life. This is what Gabbard (1990) called the loss of "basic trust", and Balint (1979) called "basic fault," while Erikson (1963) identified as the failure of the first stage of psychosocial development (basic trust versus distrust). Since, in their early years Machiavellian indiviuals were forced to experience and to build the feelings of insecurity, unpredictability, and abandonment in themselves (Láng & Birkás, 2014), therefore, they developed a kind of generalized distrust and a pessimistic attitude (Jones, 1996) towards their social environment. Their feeling of despondency over people increased, what made them susceptible to depicting others as negative, malicious and unreliable, so that they could be expecting only the worst of them. Since Machiavellian people are highly extrinsicly motivated, they also assume the same for others, which results in seeing the motivation of others much more negatively than those actually are. Machiavellianism is indeed the personality trait that is the direct opposite of trust: Gurtman (1992), Ináncsi et al. (2015) and Wrightsman (1964, 1974) found strong negative correlations between trust and Machiavellianism, while Láng (2015) in case of adolescents describes a positive relationship between Machiavellianism and distrust as early maladaptive scheme. Machiavellians' attitude of distrust accompany the expectation of negative consequences for the future (Birkás & Csathó, 2015), which also implies that they have no power to direct and control the events around them, because of their belief of external control, their lives are controlled by external forces (Jonason & Krause, 2013; McHoskey, 1999; Mudrack, 1990; Solar & Bruehl, 1971). In order to compensate for this feeling of insecurity and vulnerability caused by the loss of control, they try to control their environment and their partners in their close relationships with any possible tools (Brewer & Abell, 2017), but as control completely replaces trust (Holmes & Rempel, 1989), they will not be able to create meaningful and intimate relationships due to their inability to trust. Since the decrease of trust and the increase of the blaming attributes do not promote the development of relationships, instead, they are associated with the avoidance of social pain and protecting the self.

## Machiavellianism and Self-Protection Against Relationship Risks

Due to the grievances suffered in past relationships, Machiavellian individuals are in a state of continuous standby to protect themselves from the anticipated threats and disappointments caused by relationships. Machiavellian people find psychological intimacy especially risky, which often cause them to experience anxiety (Petrides, Vernon, Schermer, & Veselka, 2011; Richardson & Boag, 2016). Because their anxiety is mainly of social origin, Machiavellian individuals respond only to social / psychological stresses with increased reactivity, but not to physical stress (Birkás, Láng, Martin, & Kállai, 2016). The results of previous research have shown that Machiavellianism is positively related to susceptibility to paranoia (Christoffersen & Stamp, 1995; Douglas, Bore, & Munro, 2012; McHoskey, 2001a) and attachment anxiety and attachment avoidance (Gillath, Sesko, Shaver, & Chun, 2010; Ináncsi et al., 2015). Their anxiety due to social consequences is also shown by the fact that Machiavellian individuals show increased projective self-monitoring, thereby adapting their behaviour to environmental expectationsin order to avoid negative feedback of others (Rauthmann, 2011). The reason of this relation-specific social anxiety of Machiavellian individuals might be the distrust due to the acceptance and appreciation by others and the fear of rejection. This argument is supported by several studies that show that Machiavellism is positively associated with the behavioural inhibition (Jonason & Jackson, 2016; Neria, Vizcaino, & Jones, 2016). This means that Machiavellian people react to signs of punishment (and, consequently, to signs of rejection) with intensive negative emotions. The literature on rejection sensitivity (Downey & Feldman, 1996; Overall & Sibley, 2009; Pietrzak, Downey, & Ayduk, 2005) and risk regulation (Cavallo, Murray, & Holmes, 2013; Murray, Derrick, Leder, & Holmes, 2008; Murray, Holmes, & Collins, 2006) explains in detail that the anticipation and perception of rejection operates a prevention-focus motivated (Higgins, 1996, 1997) risk regulation system that is designed to detect threats as soon as possible to enable avoiding the negative-regressive states by devaluation, disapproval, insult, denial or abandonment and maintaining the feeling of security, and basically protecting the self. Insecurely attached individuals, and presumably Machiavellian people as well, react more self-protecting way to the psychological approximation, because the feelings of disappointment, insecurity and rejection represents the majority of their early interpersonal relationship experiences. They probably follow the “if-then contingency rule” (Murray et al., 2006) that “since my partner does not appreciate me and will reject me sooner or later, it is better to defend myself.” These efforts made for prevention (minimalization of risk) and keeping the distance (minimalization of dependency), however, prevents Machiavellain individuals from satisfying their basic need for belonging (Baumeister & Leary, 1995), or connecting and attaching to others (Murray et al., 2006), thereby gaining all the benefits that close intimate relationships can provide.

## Machiavellianism and Dissatisfaction With Relationships

If Machiavellian individuals do not see their partners as having desirable qualities, they are distrustful of their partners' motivation and are unable to put their self-protection aside and promote their goals of connecting, they might not experience their relationships as fulfilling, either. According to the Investment Model (Impett, Beals, & Peplau, 2001; Johnson & Rusbult, 1989; Lehmiller & Agnew, 2006; Rusbult, 1980, 1983; Rusbult & Buunk, 1993; Rusbult, Johnson, & Morrow, 1986; Rusbult, Martz, & Agnew, 1998), decrease in relationship satisfaction is accompanied by the decrease in commitment to the relationship and diminish of relationship investments, and with the upvaluation of alternative partners. Earlier studies have indeed shown that Machiavellianism, coupled with short-term mating strategy, is accompanied with low levels of intimacy and commitment (Ali & Chamorro-Premuzic, 2010; Smith et al., 2014) and - earlier examined only in case of women - the upvaluation of the perceived quality of the alternatives (Abell & Brewer, 2016).

## Machiavellianism and Gender Differences

Research on gender differences have consistently found that men have more pronounced Machiavellian traits than women (Jonason & Kavanagh, 2010; Jonason, Li, & Buss, 2010; Jonason, Luevano, & Adams, 2012; Jones & Paulhus, 2009; McHoskey, 2001b; Wilson, Near, & Miller, 1998). This may be because Machiavellian attitudes and behaviours correspond rather to men’s than to women's gender role behaviors. While men were socialized not to show their feelings and maintain their independence, women were socialized to be emotionally expressive and caring and to develop a psychological intimacy with others (Pleck & Sawyer, 1974; Taylor et al., 2000). While men are driven by the individual rewards of the relationships because of their instrumental orientation, women focus on intimacy and the progression of relationship-quality, because of their connection-oriented goals (Bekker, Bachrach, & Croon, 2007; Wright, 1982). Women are motivated to process information of intimate relationships in more detail (Acitelli & Antonucci, 1994; Tanaka, Panter, & Winborne, 1988), they also have a far more integrated approach to trust (Rempel, Holmes, & Zanna, 1985) and are more willing to see their partners and relationships in a more positive light (Murray et al., 1996b). Men, on the other hand, find psychological proximity less pleasant (Bekker, Bachrach, & Croon, 2007; Hatfield, 1984) and perceive greater risk in intimacy (Pilkington & Richardson, 1988). We expect the differences in the perceptions and attitudes of men and women with high levels of Machiavellianism of their partners and relationships to be similar to the general gender differences discussed above.

The previous theoretical overview has mainly revealed that Machiavellian behaviour is mostly driven by the negative attitudes and distrust against others, furthermore, due to the nature of their cautious disposition, also shaped by the avoidance of risks related to rejection and abandonment. To our knowledge, no study has explored these motifs of Machiavellian behaviour in case of dating couples until now. This article is unique in that respect, as it is attempting to do so.

The question is, how do individuals with high levels of Machiavellianism function in the close relationships? Does the generally negative, distrustful and cautious attitude of people with high levels of Machiavellianism prevail in the context of romantic relationships? Scholarly literature has not yet given a clear answer to these questions. Therefore, these are the questions that our article targets to unfold.

## Objectives and Hypotheses

**Hypothesis 1:** Due to the negative and cynical view on human nature (self and others) of individuals with high levels of Machiavellianism, the projection of their uncertain sense of self, their tendency to apply splitting, and higher level of neuroticism, it is expected that, not only in a general but also specifically in the context of close relationships, they look at their environment through a negative filter. Their partner and relationship perceptions and attitudes are negatively distorted.

**Hypothesis 2:** The negativity of individuals with high levels of Machiavellianism accompany a high level of distrust towards the romantic partner.

**Hypothesis 3:** Since individuals with high levels of Machiavellianism see their partners and relationships negative and untrustworthy, they are also inclined to perceive intimate relationships as dangerous and risky. This orientation prevents them to connect to the partner and increases self-protection.

**Hypothesis 4:** The defensive position of individuals with high levels of Machiavellianism leads to the deterioration of relationship satisfaction and commitment and to the decrease of investment into the relationship and increase the chances that Machiavellians seek consolation at alternative partners.

Besides investigating the relationship between Machiavellianism and partner and relationship perceptions, without formulating any hypothesis, we also aimed at exploring how Machiavellian characteristics affect the relationship perceptions and attitudes of the partner.

**Figure 1:**
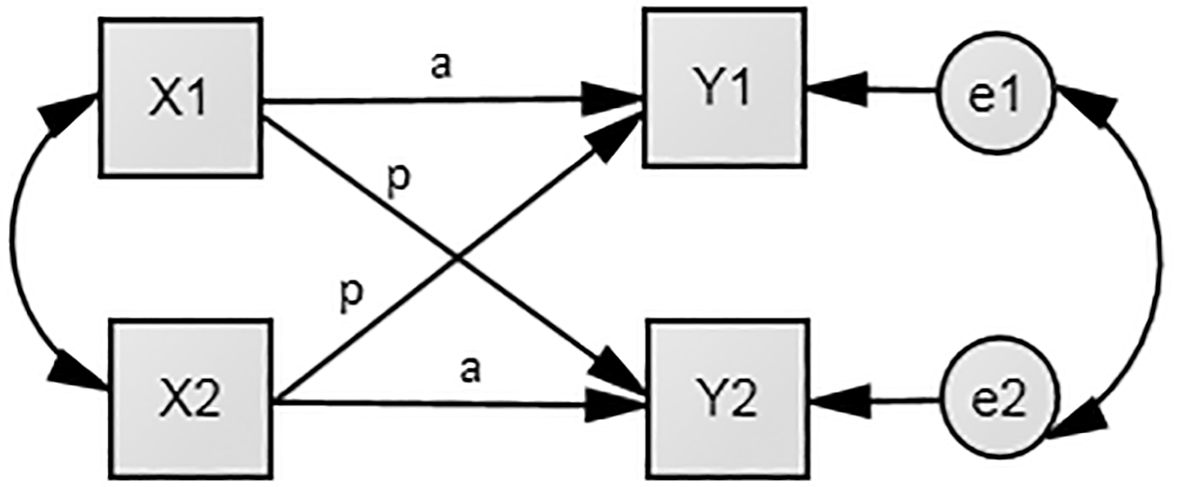
The Actor–Partner Interdependence Model (APIM), where X1 is the Machiavellianism score of men; X2 is the Machiavellianism score of women; Y1 is the score of the outcome variable for men; Y2 is the score of the outcome variable for women.

To investigate these relationsips, we used the Actor-Partner Interdependence Model (APIM; Kenny, Kashy, & Cook, 2006; Figure 1), where we could account for the effects of Machiavellianism of both parties on their own (a) and their partners’ (b) outcome variables.

Since trust is a determinative aspect of Machiavellianism, we will also examine on what variables the trust mediates the effect of Machiavellianism.

## Methods

### Participants

The sample consists of 101 Hungarian dating heterosexual couples (*N* = 202)^i^. The average age was 21.7 years (*SD* = 3.5; range: 18 - 40 years) and 23.3 years (*SD* = 4.3, range 18 - 40 years) for women and men, respectively. The length of the relationship ranged between 6 and 240 months (*M* = 30.55 months; *SD* = 31.5 months). According to the highest level of education, distribution of the sample is as follows: 4.5% of the participants finished primary school, 4.5% vocational school, 76.2% secondary school with maturity degree, and 14.9% obtained university or college degree.

### Procedure

Participants were recruited via Facebook adverts using a snowball method. Couples were volunteers. We met each couple personally on a separate occasionin the same place. In the instruction phase, they were told they would receive questionnaires with items concerning several aspects of their opinions about their partner and the relationship (e.g. their partner’s and relationship’s positive and negative characteristics etc). Prior to questionnaire completion, it was agreed that they could not know of one another´s responses since that was the only way to ensure they would respond honestly. It was assumed that if they would fill the questionnaires being aware that their answers would remain unknown to their partners, they would be more willing to disclose the negative characteristics of their partners and relationships. Both partners completed the questionnaires simultaneously but independently, seated at two separate desks. In this way, they were prevented from discussing their responses and thus the optimal conditions were ensured for data collection. Completing the questionnaires took 40 to 50 minutes. Each participant was offered 1500 Hungarian forints for their contribution (3000 HUFs – approx. 10 USDs - per couple). Participants were also informed that we would not be able to provide individual assessments since in the study we would analyse the aggregate data of 101 couples. However, participation could be beneficial to them because filling the questionnaires might help them realize several strong and weak points of their relationship for further discussion. We assured those participants who had indicated their interest that they were going to receive feedback about the major findings of the study. SPSS 21 and Amos 21 statistical softwares were used for data analysis.

### Measures

#### Machiavellianism Subscale of the Short Dark Triad Questionnaire – SD3

The scale measuring Machiavellian personality trait is a subscale of the SD3 (Jones & Paulhus, 2014). It is a 9-item, 5-point Likert scale ranging from do not agree at all (1) to agree absolutely (5). Higher scores on the scale indicate higher levels of Machiavellianism. Since internal reliability of the Mach IV (Christie & Geis, 1970) scale conventionally used to assess Machiavellianism was low for women, we omitted it from the study and only used the Machiavellianism subscale of SD3.

#### Interpersonal Quality Scale – IQS

The IQS (Murray, Holmes, & Griffin, 1996a, 1996b) measures global attitudes towards the partner. It is an internally consistent, two-factor scale consisting of 21 highly precise items, 12 measuring the positive and 9 the negative partner attitudes on 8-point Likert scales ranging from not at all (1) to absolutely (8). Altough the original IQS did not included it, in order to clarify the purpose of measurement, both groups of items are introduced with the following sentence: *“Considering only the positive/negative characteristics of your partner and ignoring the negative/positive ones, evaluate how positive/negative these characteristics are. My partner is...”* This enables participants to evaluate their partners’ positive and negative qualities separately. Each quality is rated on an unipolar scale, on which higher scores represent either more positive or more negative evaluation of the partner. The positive and negative indicators can also be conceptualized as estimations of satisfaction with the partner. More positive attitudes reflect higher satisfaction while more negative attitudes reflect higher levels of dissatisfaction with the partner.

#### Positive and Negative Semantic Differential – PNSD

The PNSD (Mattson, Rogge, Johnson, Davidson, & Fincham, 2013) measures global attitudes toward the relationship. It is an internally consistent, two-factor scale consisting of 14 highly precise and informative semantically differentiating items, 7 measuring the positive and 7 the negative relationship attitudes on 8-point Likert scales ranging from not at all (1) to absolutely (8). Both groups of items are introduced with the following sentence: *“Considering only the positive/negative characteristics of your relationship and ignoring the negative/positive ones, evaluate how positive/negative these characteristics are. My relationship is...”* This enables the participants to evaluate their relationships’ positive and negative qualities separately. Each quality is rated on an unipolar scale, on which higher values represent either more positive or more negative evaluation of the relationship. The positive and negative indicators can also be conceptualized as estimations of satisfaction with the relationship. More positive attitudes reflect higher satisfaction, while more negative attitudes reflect higher levels of dissatisfaction with the relationship.

#### Scales of Risk Regulation System

The Risk Regulation Sytem (Murray, Derrick, Leder, & Holmes, 2008) is a dual operation process that regulates switching between connectedness goals and self-protection goals according to the perceived safety or risk of the particular relationship context.

##### Connectedness goals

This scale measures the focus on the positive, desirable and rewarding aspects of the relationship, which is a definitive approach orientation. It is a 7-item, 9-point Likert scale ranging from not at all true (1) to absolutely true (9). Higher scores indicate more optimistic views of the relationship.

##### Self-protection goals

This scale measures the focus on the negative, threatening and risky aspects of the relationship, which involves avoidance of such costly situations as well as self-defence. It is a 10-item, 9-point Likert scale ranging from not at all true (1) to absolutely true (9). Higher scores indicate that connectedness is perceived as more threatening and risky.

#### Investment Model Scale – IMS

We employed the original IMS (Rusbult, Martz, & Agnew, 1998). The scale comprises 37 items, 15 of which are facet items assessed on a 4-point Likert scale, while 22 are global items assessed on a 9-point Likert scale (point 1 representing absolute disagreement and point 4 or 9 indicating absolute agreement, respectively). Since in this assessment the facet items only contribute to a better interpretation of the global items, only the global items were aggregated. The scale has four subscales:

##### Satisfaction

A subscale consists of 5 facet items and 5 global items and measures the satisfaction with the partner and the relationship.

##### Quality of alternatives

A subscale consists of 5 facet items and 5 global items and measures the extent to which a respondent’s needs may be fulfilled in alternative relationships (e.g. a different partner, friends, family) to their current partner and how attractive they find alternative partners.

##### Investment

A subscale consists of 5 facet items and 5 global items and measures the amount of investment of intrinsic resources (e.g. emotions, self-disclosure, time) and extrinsic resources (e.g. financial resources, mutual activities, mutual friends) in the relationship.

##### Commitment

A subscale consists of 7 global items and measures commitment to the partner and the relationship.

#### Trust in Close Relationship Scale – TCL

The Trust in Close Relationship Scale (Rempel, Holmes, & Zanna, 1985) has been specifically designed to tap how much the participants trust their partners in the close relationships. It is a 17-item, 7-point Likert scale ranging from do not agree at all (1) to agree absolutely (7). Higher scores indicate higher trust in the partner. The scale has three subscales:

##### Predictability

A 5-item subscale measuring expectations on the partner’s future behaviour.

##### Dependability

A 5-item subscale measuring trust in the partner's specific internal qualities as the relationship develops.

##### Faith

A 7-item subscale measuring unconditional trust in the partner.

## Results

### Descriptive Statistics

Table 1 shows the means, standard deviations and internal realiability of each scale for men and women separately. We analysed gender differences by paired samples t-tests. The results indicate that men are more Machiavellian than women: they view their partners and relationships in a more negative and less positive light, they are less committed to their relationships, trust their partner less, perceive their partners less predictable, and are more open to alternative partners.

**Table 1:** Reliabilities, Means, Standard Deviations, and Paired T Tests by Sex for Machiavellianism and Key Variables

Measure	Dating Sample						*t*	*p*
	Males (*n* = 101)		*IR*	Females (*n* = 101)		*IR*		
	*M *	*SD*		*M*	*SD*			
SD3 Mach	3.25	0.68	.74	2.89	0.65	.69	4.209	˂ .001
IQS-POS	6.31	0.73	.78	6.48	0.73	.76	-1.831	˂ .050
IQS-NEG	3.14	1.06	.77	2.72	0.89	.70	3.048	˂ .005
PNSD-POS	6.71	0.92	.85	6.94	0.81	.83	-2.550	˂ .050
PNSD-NEG	1.64	0.71	.82	1.35	0.57	.82	3.430	˂ .005
Connectedness Goals	6.82	1.03	.63	7.04	1.04	.62	-1.679	.096
Self-Protection Goals	3.98	1.52	.83	4.06	1.78	.88	-0.390	.697
Quality of Alternatives	3.89	1.65	.77	3.35	1.60	.79	2.513	˂ .050
Investment	6.36	1.52	.73	6.35	1.61	.74	0.069	.945
Satisfaction	7.80	1.22	.90	7.79	1.21	.88	0.046	.963
Commitment	8.34	1.08	.89	8.62	0.84	.89	-2.388	˂ .050
Trust	5.50	0.88	.88	5.71	0.76	.83	-2.245	˂ .050
Predictability	4.82	1.04	.68	5.21	0.98	.67	-3.169	˂ .005
Dependability	5.72	1.16	.74	5.81	1.10	.72	-0.582	.562
Faith	5.97	0.86	.82	6.09	0.81	.80	-1.353	.179

In order to measure nonindependence with distinguishable dyad members and interval level scores the members scores were correlated. The correlations between the scales were measured by Pearson coefficients, as shown in Table 2. Here we see that scores of men and women are moderately correlated on the scales for positive relationship attitudes (PNSDPOS), satisfaction, relationship trust and faith, and weakly correlated on the other scales (e.g. for commitment, predictability). The correlations of the partners' scores indicate that these are not independent observations, therefore in our analysis we must use the dyad as an analytical unit.

**Table 2:** Intercorrelations (Pearson´s) Among Key Variables by Gender (Men Below Diagonal, Women Above Diagonal, Correlations Between Men and Women are on the Diagonal in Parentheses)

Measure	SD3 Mach	Interpersonal Quality Scale		Positive and Negative Semantic Differential		Risk Regulation System		Investment Model Scale				Trust in Close Relationship Scale			
	1	2	3	4	5	6	7	8	9	10	11	12	13	14	15
1. SD3Mach	(.16)	-.08	.06	-.09	.05	.09	.19	-.06	.06	.22*	.09	-.21*	-.20*	-.13	-.16
2. IQSPOS	-.09	(.15)	-.50**	.62**	-.39**	.30**	-.12	.50**	-.25*	.16	.34**	.58**	.34**	.47**	.61**
3. IQSNEG	.35**	-.51**	(.04)	-.28**	.29**	-.07	.26**	-.33**	.18	.08	-.10	-.43**	-.45**	-.30**	-.30**
4. PNSDPOS	-.08	.61**	-.45**	(.46**)	-.58**	.34**	-.25*	.72**	-.29**	.13	.41**	.48**	.23*	.34**	.58**
5. PNSDNEG	.21*	-.50**	.60**	-.65**	(.17)	-.32**	.36**	-.77**	.29**	-.03	-.57**	-.33**	-.16	-.16	-.46**
6. Connect Goal	-.05	.25*	-.21*	.47**	-.34**	(.18)	.10	.34**	-.17	.29**	.24*	.01	-.16	.07	.13
7. SelfProt Goal	.27**	-.27*	.32**	-.32**	.43**	.05	(.19)	-.36**	.15	.10	-.28**	-.40**	-.47**	-.22*	-.27**
8. SAT	-.09	.55**	-.39**	.70**	-.66**	.49**	-.31**	(.43**)	-.36**	.05	.58**	.58**	.34**	.40**	.66**
9. QOA	.30**	-.29**	.34**	-.25*	.35**	-.29**	.17	-.38**	(.13)	-.17	-.45**	-.25*	-.11	-.27**	-.24*
10. INV	-.05	.00	-.04	.19	-.25*	.42**	.11	.27**	-.22*	(.16)	.24*	.00	-.12	.08	.07
11. COM	-.09	.24*	-.15	.32**	-.32**	.62**	-.17	.54**	-.45**	.38**	(.24*)	.34**	.13	.18	.48**
12. Trust	-.26**	.51**	-.55**	.58**	-.58**	.37**	-.50**	.57**	-.25*	.16	.30**	(.36**)	.81**	.82**	.81**
13. Predict	-.24*	.38**	-.51**	.44**	-.46**	.24*	-.48**	.43**	-.17	.07	.17	.90**	(.26*)	.53**	.40**
14. Depend	-.24*	.43**	-.47**	.53**	-.56**	.35**	-.44**	.53**	-.19	.14	.28**	.84**	.64**	(.12)	.54**
15. Faith	-.23*	.53**	-.48**	.56**	-.53**	.39**	-.39**	.57**	-.29**	.21*	.35**	.90**	.70**	.65**	(.38**)

*p < .05. **p < .01.

Between the SD3Mach used as predictor variables and output variables (IQSNEG, PNSDNEG, self-protection, quality of alternatives, trust and its free aspects) in the analyses, we can find consistent correlation with the theoretical summary rather in case of men, while we have fewer and weaker correlations case of women (e.g. investment, trust and predictability).

The questionnaires have adequate convergent and discriminative validities. Most the correlations are in the expected direction, and are statistically significant for both sexes. For example, trust and satisfaction correlates positively with positive partner and relationship attitudes (IQSPOS, PNSDPOS) and negatively with negative partner and relationship attitudes (IQSNEG, PNSDNEG) in both sexes. This confirms the idea suggested in the hypothesis that more negative attitudes are associated with greater distrust and dissatisfaction. Self-Protection correlates positively with negative partner and relationship attitudes (IQSNEG, PNSDNEG) and negatively with satisfaction and trust in both sexes. These results show that the negative partner and relationship attitudes are associated with stronger self-protection and less satisfaction and trust. While it only applies to men with high levels of Machiavellianism, it is still a suggestive finding that negative partner attitudes (IQSNEG) are positively correlated to the quality of alternative partners, which means that in parallel with increasing devaluation of the qualities of their partners, Machiavellian men attribute greater significance to alternative partners.

Although the positive and negative attitude scales of the IQS and PNSD are theoretically independent dimensions (Berntson, Boysen, & Cacioppo, 1993; Costa & McCrae, 1980; Tellegen, 1985; Watson & Clark, 1997), our findings show they are interrelated: we found moderate negative correlations between these scales.

### Actor-Partner Interdependence Model (APIM)

We examined the relationship between Machiavellianism and the different relationship components with Actor-Partner Interdependence Model (APIM – Kenny, Kashy, & Cook, 2006). This provided a picture of both the actor effects (the effects of the respondent’s Machiavellianism score on the output variables) and the partner effects (the effects of partner’s Machiavellianism score on the respondent’s output variables). The models were analyzed with structural equation modeling where maximum likelihood estimation were carried out. 14 individual models were estimated, one for each outcome variables, the models were just-identified (Table 3).

**Table 3:** APIM for Machiavellianism (SD3 Machiavellianism) and Relationship Key Variables

Key Variables	Coefficient (Beta)				Unstandardized Estimate
	Actor Mach (man)	Partner Mach (woman to man)	Actor Mach (woman)	Partner Mach (man to woman)	Covariance between Outcome Errors
IQSPOS	-.072 (-.067)	-.180 (-.161)	-.081 (-.073)	-.051 (-.048)	.067
IQSNEG	.503 (.322)***	.269 (.165)	.091 (.067)	-.047 (-.036)	.032
PNSDPOS	-.081 (-.060)	-.181 (-.128)	-.110 (-.088)	.009 (.007)	.334***
PNSDNEG	.205 (.187)*	.061 (.057)	.032 (.037)	.048 (.057)	.061
Connectedness Goals	-.058 (-.038)	-.148 (-.093)	.135 (.085)	.020 (.013)	.201
Self-Protection Goals	.592 (.263)**	.150 (.064)	.554 (.203)*	-.176 (-.067)	.496
Quality of Alternatives	.738 (.304)**	-.126 (-.050)	.132 (.054)	.120 (.051)	.303
Investment	-.134 (-.060)	.073 (.032)	.636 (.257)**	-.517 (-.218)*	.339
Satisfaction	-.148 (-.083)	-.050 (-.027)	-.099 (-.054)	-.054 (-.030)	.625***
Commitment	-.156 (-.098)	.103 (.062)	.150 (.116)	-.192 (-.155)	.200*
Trust	-.288 (-.224)*	-.347 (-.258)**	-.234 (-.200)*	-.050 (-.045)	.190**
Predictability	-.295 (-.194)*	-.427 (-.269)**	-.320 (-.213)*	.092 (.064)	.215*
Dependability	-.356 (-.209)*	-.333 (-.188)	-.200 (-.119)	-.071 (-.044)	.112
Faith	-.243 (-.193)*	-.268 (-.218)*	-.179 (-.143)	-.160 (-.134)	.214**

*Note.* Estimate between men’s and women’s Machiavellianism = .077. *N* = 101 couples.

*p < .05. **p < .01. ***p < .001.

- For men, Machiavellianism (SD3) was found to have significant positive actor effects on the negative subscales of the partner and relationship attitude questionnaires (IQSNEG, PNSDNEG), as well as on self-protection and the quality of alternatives. These findings indicate that highly Machiavellian men perceive their current partners and relationships more negatively, pursue more self-protection goals in their close relationships and have a more pronounced tendency to seek alternative partners. Furthermore, men’s Machiavellianism (SD3) also showed significant, but negative effects on relationship trust as well as on each of its three aspects (Predictability, Dependability, and Faith). These results show that highly Machiavellian men trust their partners less (perceive their partners less predictable, less dependable, and have less faith in their partners).
- With regard to woman to man partner effects, our finding is that the women’s Machiavellianism has significant or marginally significant negative effects on their male partners’ trust and on its two aspects (Predictability and Faith). Thus, women with higher levels of Machiavellianism have male partners who perceive them as less trustworthy in general, less predictable and with less faith in the relationship in detail.
- For women, Machiavellianism (SD3) was found to have significant positive actor effects on self-protection and relationship investment. These findings indicate that highly Machiavellian women pursue more self-protection goals in their intimate relationships, and they also invest more into their relationships. Furthermore, women’s Machiavellianism (SD3) also showed significant, but negative actor effects on relationship trust and predictability. These results show that highly Machiavellian women trust their partners less and perceive them as less predictable.
- Regarding man to woman partner effects, we have found that men’s Machiavellianism only affects the level of women’s investment, indicating that the female partners of highly Machiavellian men invest less in their relationships.

We have also found some contrast effects. For trust, predictability and faith of men with high levels of Machiavellianism, the actor and partner effects are in the same direction, while for investment of women with high levels of Machiavellianism, the actor and partner effects are in the opposite direction. This latter contrast effect indicates that the women’s own Machiavellianism increases their investment in the relationship while their male partners’ Machiavellianism reduces it.

Subsequently, we tested various mediaton models to analyse the relationship between Machiavellianism and the different relationship components. The obtained results revealed that trust has a prominent role since it mediates the effect of Machiavellianism on several variables. For instance, men’s trust mediates the effects of both men’s and women’s Machiavellianism on men’s negative partner and relationship perceptions [IQSNEG: men (*b* = .107; *p* = .025), women (*b* = .142; *p* = .005); PNSDNEG: men (*b* = .131; *p* = .025), women (*b* = .166; *p* = .006)], self-protection goals [men (*b* = .104; *p* = .016), women (*b* = .128; *p* = .007)] and relationship investments [women (*b* = -.067; *p* = .052)]. Machiavellianism in women influences women’s level of trust, which in turn affects men’s openness towards alternative relationships (*b* = .088; *p* = .019).

**Figure 2:**
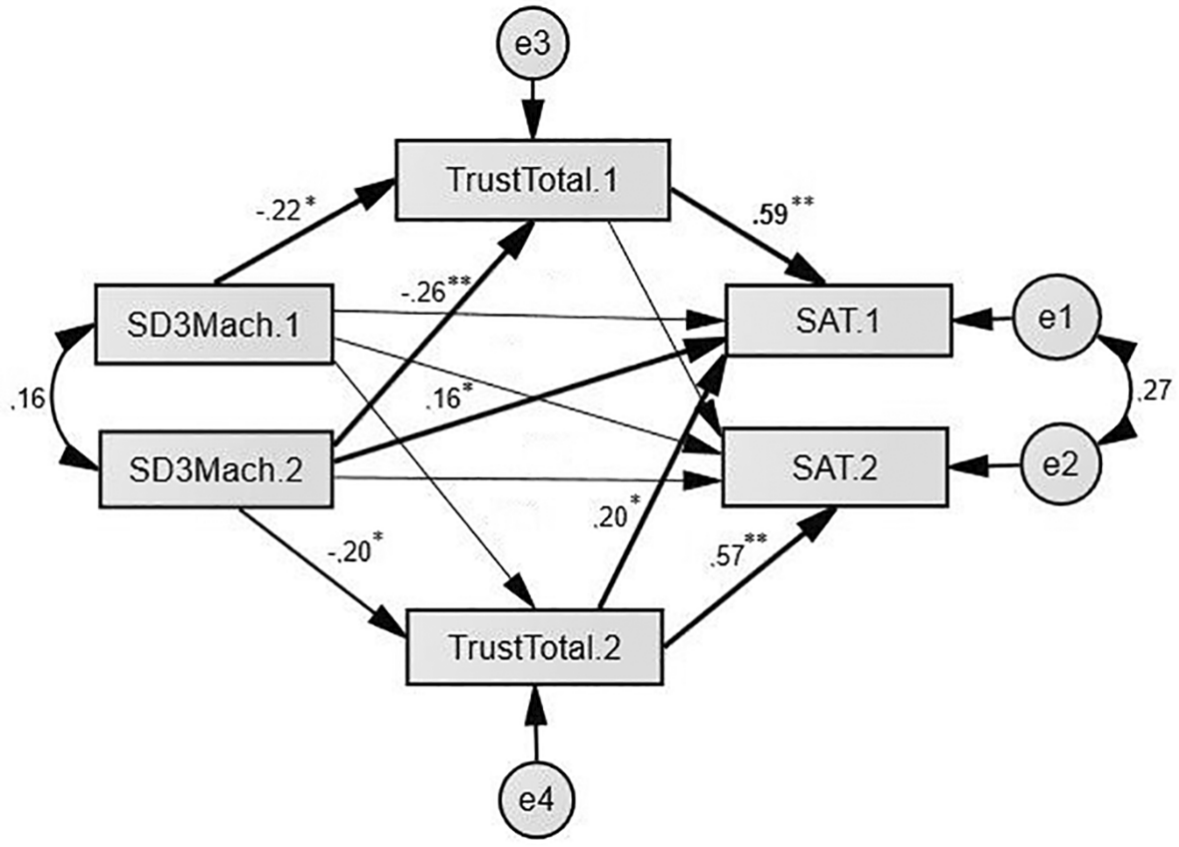
APIMeM showing Trust mediating the path between Machiavellianism and Satisfaction. Values are standardized regression coefficients and correlations. „e” represent the error term for each variable. SD3Mach (Machiavellianism); Trust (TCL); Satisfaction (IMS) (Men = 1; Women = 2). *p < .05. **p < .01.

We have also found trust to play a mediating role on satisfaction (Figure 2). There are three significant indirect paths (equivalent to the drop in the direct path between Machiavellianism and Satisfaction when the mediating variable was controlled):

SD3Mach1 – Trust1 – SAT1 (*b* = -.140; *p* = .033);

SD3Mach2 – Trust2 – SAT2 (*b* = -.147; *p* = .021);

SD3Mach2 – Trust1 – SAT1 (*b* = -.191; *p* = .004).

These results show, that higher Machiavellianism predicted lower trust, wich in turn fed into more negative perceptions of relationship satisfaction (Model fit: *p* (Chi2) = .001; CFI = .918; RMSEA = .279).

## Discussion

### Negativity

The purpose of this study was to analyse whether the negative, distrustful and cautious attitude of people with high levels of Machiavellianism prevail in the context of close relationships, and what effect their pessimistic tendencies have on the other consequences of their relationship (satisfaction, commitment, investment, quality of alternatives). From the results of previous studies (e.g. Kraut & Lewis, 1975; Láng, 2015; Láng & Lénárd, 2015; Ojha, 2007; Touhey, 1973), it can be concluded that the emphasis is on the toxic effect [(e.g. "basic fault", Balint, 1979)] of the early socialization environment rather than on its modelling effect in developing the Machiavellian personality, which then exerts far-reaching effects on the individual’s life and adult relationships. The representations of their past conflictual object relations are kept alive in their subjective world even in adult age, which penetrates into their present relationships distorting the authentic observation of their partners and relationships. It is logical to think that the negative partner and relationship attitudes of highly Machiavellian individuals are the result of perceiving through this dark prism. Indeed, the results of the correlations and actor effects of the present study show that men with high levels of Machiavellianism perceive both their partners and their relationships in a negative light. These findings support most part of Hypothesis 1. Although women with high levels of Machiavellianism appear to be less negativistic, this is likely to be caused by some moderating variables [e.g. the need to belonging and psychological intimacy (Pleck & Sawyer, 1974; Taylor et al., 2000)] that reduce the expression of negativity and its distorting effect on perception in case of Machiavellian women. It is a fact that highly Machiavellian women do not see their partners or their relationships positively, either.

### Distrust

If persons with high levels of Machiavellianism perceive their partners negatively, supposedly they also see them untrustworthy. The correlation and actor effect results consistently with one another indicatet that men and women with high levels of Machiavellianism show considerable distrust towards their partners. Only in three aspects of trust are there some differences between the sexes. Men and women with high levels of Machiavellianism both regard their partners less predictable (predictability), but men are also less likely to associate them with positive qualities (reliability) and they are also reluctant to rely unconditionally on their partners (faith). These results are in line with the traditional gender roles that men generally experience intimacy more risky than women do (Pilkington & Richardson, 1988) and women have a more integrated view of trust (Holmes & Rempel, 1989), they are more willing to see positive features in their partners, to positively idealize the partners (Murray et al., 1996b), and to believe that, whatever the future holds, their partner will be loving and caring (Rempel, Holmes, & Zanna, 1985). These results supported Hypothesis 2. In the case of distrust, the partner effects show that while women’s Machiavellianism significantly and negatively predicted men's trust and two aspects of trust (predictability and faith), mens' Machiavellianism do not predict women's trust. The less negative and less distrustful attitude of highly Machiavellian women to Machiavellian men is partly explained by the fact that Machiavellian male partners may be attractive for women in some ways. We can reason that for a woman and her children a Machiavellian male partner may mean evolutionary (survival) advantage, if he uses his manipulative skills against the external social environment in the favour of his family's assets (for example a Machiavellian partner can be more successful in obtaining the material resources due to his external orientation.) Indeed, there are studys showing that women are attracted to Machiavellian men, to the “bad guys” (Aitken, Lyons, & Jonason, 2013; Carter, Campbell, & Muncer, 2014).

### Self-Protection

Since individuals with high levels of Machiavellianism see their partners and relationships as negative and untrustworthy, therefore, they perceive close relationships dangerous and risky, which reduces Machiavellians’ goals to reach relationship reward (promotion focus) and enhances their levels of cautiousness and defenses (prevention focus) against anticipated threats (Jonason & Jackson, 2016; Neria et al., 2016). The actor effects and partially also the correlations, indeed, indicate that both men and women with high levels of Machiavellianism show a self-protective orientation in their close relationships, the main purpose of which is to avoid negative-regressive states entailing devaluation, rejection, or abandonment. This means that, by anticipating negative consequences, they are trying to minimize the pain of vulnerability and rejection by all possible ways. In order to achieve this, they (especially highly Machiavellian men) apply cognitive and behavioral strategies that are intended to decrease dependency on the partner and increase emotional distance (Levinger, 1979; Murray et al., 2006; Murray, Holmes, & Griffin, 2000). Such cognitive strategies are the devaluation of the partner's qualities (e.g. negative partner attitudes) (Kernberg, 1975; Richardson & Boag, 2016), blame of the partner (Rasmussen & Boon, 2014), and the devaluation of the importance of connecting and intimacy (e.g. negative relationship attitudes) (Ali & Chamorro-Premuzic, 2010). To reduce dependency, they use a behavioral strategy that they stay away from their actual partner not only emotionally but also physically – thus limiting the situations where the partner can be disapproving – and they seek support from alternative partners. The research from Abell and Brewer (2016) supported the preference of external partners in case of women, while the current study found the same in the case of men and women. This result also raises the possibility that the Machiavellian individuals might flee from the anticipated rejection to ever-newer alternative partners. This support Hypothesis 3.

### Dissatisfaction (Commitment, Investment, Quality of Alternative Partners)

One particularly important question is what is the impact of the highly pessimistic attitude of the individuals with high levels of Machiavellianism on the long-term implications of the relationship, such as satisfaction, commitment and investment (Rusbult et al., 1998)? The results of the mediation models (Figure 2) demonstrate that distrust is a central and predictive factor in Machiavellian personality, wich fed into more negative perceptions of relationship satisfaction. Several authors emphasized the importance of optimism and positive emotions and attributions for the relationship well-being (Murray et al., 1996a). It has been shown that for satisfaction and commitment, the ratio of positive interactions to negative interactions has to be at least 5:1 (Byrne & Murnen, 1988; Gottman, 1994; Gottman & Levenson, 1992). For highly Machiavellians, the accumulation of negative attributes is likely to deteriorate the relationship satisfaction. Some authors (e.g. Fincham & Linfield, 1997; Mattson et al., 2013) have already conceptualized positive and negative partner and relationship attitudes as indicators of relationship satisfaction or dissatisfaction. Based on this, the results of partner and relationship attitudes (IQS, PNSD) show that men with high levels of Machiavellianism are dissatisfied with their partners and their relationships, as well (Table 2, Table 3). Although women with high levels of Machiavellianism show no romantic satisfaction either, however, the results show that they are less dissatisfied with their Machiavellian male partners, which is consistent with the existing studies (Smith et al., 2014).

While commitment in this study has not indicated any link between Machiavellism in case of men and women (in contrast with other researches, e.g. Ali & Chamorro-Premuzic, 2010), however, investments in the relationship in case of female Machiavellianism and the quality of the alternatives in case of male Machiavellianism presented significant correlation. These results are in line with the Investment Model (Rusbult, Martz, & Agnew, 1998), what explains that the decrease in relationship satisfaction is accompanied by an increase in the perceived quality of alternative partners (Felmlee, Sprecher, & Bassin, 1990; Johnson & Rusbult, 1989). Indeed, men with high levels of Machiavellianism find alternative partners more desirable than their current partners. We might speculate that as quality of their partners decreases, so does the appreciation of the quality of their alternative partners increase.

Although we did not formulate any expectations regarding that, a much more interesting result emerged. For women, relationship investment are related to both their own and their partner’s Machiavellianism. A contrast effect can be observed here where women's own Machiavellism increases their investment into the relationship, while the Machiavellism of their male partners reduces that. According to the Investment Model, the more an individual prefers to stay in a relationship, the more willing the individual is to invest into the relationship (Lehmiller & Agnew, 2006). This means that for women with high levels of Machiavellianism, self-protection does not completely exclude the search for connectedness goals (Jonason, Li, Webster, & Schmitt, 2009). From previous results (Ináncsi et al., 2016), we also know that Machiavellians live in externalized relationships where couples are typically bound together not by intrinsic investments (e.g. warmth, empathy and self-disclosure) but rather the extrinsic rewards (e.g. acquisition of resources, status and sex). At first glance, highly Machiavellian women may seem to want to reach their partner's increasing commitment and the reduction of their partner's interest in alternatives through the use of their investments in the relationship. However, we can also speculate that the investment of women with high levels of Machiavellianism in the relationship is a means of power, control and manipulation over the partner. As it is well known, there are serious calculations behind the facade of Machiavellian “love”. Highly Machiavellian women may be aware that the desire for extrinsic rewards is one of the weakest points of Machiavellian men. Machiavellian women generally have fewer opportunities to use harder, more direct influencing tactics, like pressure by physical violence, in manipulating their male partners. Thus they rather prefer more delicate, soft tactics instead, for example, by investing in the relationships through ingratiating, friendliness, charm, or flattery, which are rather indirect and are based on emotional influences. In case of women, investments can certainly also be of instrumental nature in tactics to gain personal benefits and rewards such as exchanging or winning a partner's alliance, which involves reciprocity. If softer tactics are not successful, women's investments may become a device of coercion. If investments make a better bargaining position against male partners, manipulative women can easily position themselves in order to blackmail their obliged (and not committed) male partners. The investments can become a device of power and of blackmailing as the relationship progresses. Machiavellian women, as being good Machiavellians, first use soft tactics, and if they do not succeed (for example, if the male partner is manipulative and exploitative himself), only then do they apply harder tactics to control and keep a firm hand on their male partners (Reimers & Barbuto, 2002). In this case, investments of women with high levels of Machiavellianism are a form of passive aggression (Grams & Rogers, 1990). Our hypothesis 4 is mostly confirmed in case of man.

## General Conclusion

The general conclusion is that people with high levels of Machiavellianism are not able to overcome their negative, distrustful, and cautious attitudes even in their close relationships. In a risky venture like intimate relationships, Machiavellian individuals feel particularly vulnerable and exposed. Since they - especially highly Machiavellian men - are stuck in every bad self-object-state of splitting (bad self - bad object), they seem to be unable to develop healthy object-relationships. In their fear of being abandoned and rejected again - as it happened in their childhood -, Machiavellian individuals focus strongly on the negatives and on the losses. They can not trust the honest appreciation and commitment of their partners, so they place self-protection ahead of relationship promotion. This pessimistic attitude, however, undermines the well-being of the relationships and prevents them from establishing and maintaining intimate and truly satisfactory interpersonal relationships.

## Limitations and Future Directions

The main limitation of the study is its cross-sectional design, namely that relationship attitudes and perceptions were examined only at one given point in time. As evaluations of the relationships can change continuously over the time, a longitudinal study should explore the development and variation of relationship attitudes and perceptions. When analysing the system of connections, it would be fortunate to examine the relationship of Machiavellism with the examined relationship characteristics and control variables within one model, however, this is not possible because of the number of elements in the sample. Although compared to the typical sample size of a dyadic research our sample can be considered average sized, it is not suitable for analysing complex models.

Is it still an important research question that how does Machiavellism affect the relationship quality and the long-term development of the relationship? Our article was an initial attempt to highlight the conflicting relationship between Machiavellianism and relationship satisfaction, but the task remains for a future research to explore the different paths Machiavellianism takes in men and women in exerting its destructive effects on close relationships.
